# Physical Activity in Adolescents Living in Rural and Urban New Caledonia: The Role of Socioenvironmental Factors and the Association With Weight Status

**DOI:** 10.3389/fpubh.2021.623685

**Published:** 2021-08-06

**Authors:** Guillaume Wattelez, Stéphane Frayon, Corinne Caillaud, Olivier Galy

**Affiliations:** ^1^Interdisciplinary Laboratory for Research in Education, University of New Caledonia, Noumea, New Caledonia; ^2^Faculty of Medicine and Health, Charles Perkins Centre, School of Medical Sciences, University of Sydney, Sydney, NSW, Australia

**Keywords:** exercise, sedentarity, lifestyle, Melanesian, Polynesian, Pacific, obesity, sitting time

## Abstract

Physical activity (PA) is an important factor for the prevention of overweight and obesity, particularly during adolescence. This study focuses on the understudied adolescent population of New Caledonia with the aim to (1) determine the daily PA levels and estimate the sedentary time through out-of-school sitting time; (2) highlight the influence of sociodemographic and environmental factors, and (3) assess the associations of PA and sitting time with overweight and obesity. A sample of 508 school-going adolescents living in New Caledonia was surveyed about their PA habits using the International Physical Activity Questionnaire–Short Form, as well as about the context in which they usually engage in PA. The influences of the place of living and ethnic community were also investigated. Results indicated that about 66% of the adolescents performed an average of at least 60 min of PA daily. Both Melanesian adolescent boys and girls were more active than Caucasian adolescents but only when they lived in rural areas (females: 115 vs. 93 min/day, *p* = 0.018; males: 133 vs. 97 min/day, *p* = 0.018). Indeed, PA was reduced in an urban environment (females: 88 min/day; males: 95 min/day, *p* = 0.028; rural vs. urban in Melanesian adolescents). Melanesian adolescents also spent less time in out-of-school sitting than Caucasian adolescents independently of where they lived (females: 164 vs. 295 min/day, *p* < 0.001; males: 167 vs. 239 min/day, *p* = 0.001). Feeling safe was positively associated with PA levels (females: OR_adj_ = 2.85, *p* < 0.001; males: OR_adj_ = 4.45, *p* < 0.001). In the adolescent boys, accessibility to a suitable place was also an important factor (OR_adj_ = 2.94, *p* = 0.002). Finally, while PA and sitting time were negatively associated with overweight in male adolescents (OR_adj_ = 0.28, *p* = 0.044 and OR_adj_ = 0.39, *p* = 0.004), they were not in females. Living in a rural area allowed the Melanesian adolescents to maintain a more active lifestyle with more physical activities and less sitting time. Our results also indicated that safety was an important driver for engagement in PA. The urban environment in New Caledonia appears to be a contributor of a less active lifestyle in adolescents.

## Introduction

Many factors are related to obesity, including gender, ethnic background, income level and educational background (1–3). Lifestyles and habits are also related to the development of overweight and obesity, particularly increased fast food and soft drink consumption, frequent dieting attempts, low physical activity (PA), and long hours watching television (1, 4). Sedentary time, which is a major risk factor for non-communicable diseases, has increased worldwide in the past few decades (5, 6). In addition, and despite the health benefits of PA (7), adolescents around the world do not meet the current guidelines (5, 8–11). Yet in children and adolescents, PA confers such benefits as improved physical fitness (including both cardiorespiratory and muscular fitness), cardiometabolic health (improved control of blood pressure, dyslipidemia, glucose, insulin resistance), bone health, cognitive outcomes (academic performance, executive function), and mental health (reduced symptoms of depression), and it is favorably associated with adiposity (12). While doing some PA is better than doing none, these benefits require an average of at least 60 min per day of moderate- to vigorous-intensity PA (MVPA) across the week (13, 14). This means that <60 min/day of MVPA would be considered as insufficient even if an individual was active all day in the course of professional activity. Conversely, someone having high sedentary time because of professional activity (in an office, for instance) could be considered as physically active provided that he/she has practiced at least 60 min/day of MVPA (13). Similarly, a schooled adolescent who is seated all day long in school can have sufficient MVPA time (before or after school times) to be considered physically active. Notably, sedentary behavior is often associated with unhealthy habits such as snacking and high screen times (4).

The social environment, which can influence health behaviors like PA and sedentary time, has received increasing attention in recent years (2, 3, 15–18). Influences from family and friends can occur through social pressure, social modeling and imitation, social comparison, and behavior approximation (8, 15, 19–22). For example, studies have highlighted that parental modeling and parental support may be associated with the child's and adolescent's PA (22–24). Friends may be more influential than other social contacts, however, because of a clearer understanding of the information coming friends, resulting in better internalization (16, 25, 26). Although social influences on PA occur throughout life, they are particularly important during adolescence, especially since behavior acquired at a young age can determine behaviors and lifestyle choices into adulthood (8, 23).

In the Pacific region, islanders have increasingly adopted Western modes of living in the past few decades (27, 28). This has caused fundamental changes in lifestyles and a dramatic increase in non-communicable diseases (29). Globalization, trade liberalization and increasing urbanization have all contributed to shifts in PA and diet, leading to a steadily increasing prevalence of overweight (30–35). The Polynesian and Melanesian populations of the French territories in the Pacific are particularly exposed to lifestyle Westernization (36–39). New Caledonia has the particularity of approaching the economic level of Western countries due to industrial and mining activities. While some Caledonians live in cities and have adopted a Western lifestyle, others still live a more traditional Pacific lifestyle, which is characterized by fishing, agriculture and cultural activities that follow traditional customs (e.g., house construction, weddings, mourning, customary ceremonies) and is generally associated with high daily PA (40). Conversely, Oceanian adolescents living in urban areas may adopt a more sedentary lifestyle (32, 41) that favors obesity and its health consequences. In New Caledonia, Zongo et al. investigated Melanesian adolescents' physical fitness, PA and body composition using questionnaires and anthropometric measures (42). They found that Melanesian adolescents living in rural environments had good physical fitness, were more active, and had a higher percentage of body fat than Melanesian adolescents living in urban environments, especially boys (42). However, from a pilot study using wrist activity trackers, Galy et al. found that rural Melanesian adolescents performed only ~30 min of moderate to vigorous PA per day on average, which is half the World Health Organization (WHO) recommendations (11, 43). Although specific health education programs are needed in the schools of New Caledonia, as in other Asia-Pacific countries (44), little is known about the patterns of PA in this region and the reasons why certain people adopt or maintain active lifestyles whereas others do not (42). Moreover, studies have suggested that financial limitations, family commitments, time constraints, and road safety issues restrict healthy lifestyle behaviors in the Melanesian population from Vanuatu, especially for women (45–47).

Several studies conducted in a range of countries have identified ethnicity as a factor of PA levels (2, 19, 23, 48, 49), while others have found no consistent relations (50). In any case, it is not yet known whether ethnicity is associated with PA levels in the adolescents from the multicultural population of New Caledonia. We thus hypothesized that living in rural areas with a traditional Melanesian lifestyle would be associated with higher PA levels and less sedentary time.

This study aimed to (i) assess the time that New Caledonian adolescents spend in PA and in a sitting position, (ii) analyze the possible associations between PA and sitting time and the sociodemographic or environmental characteristics, and (iii) identify the associations of PA and sitting time with overweight in these adolescents.

## Materials and Methods

### Data Collection and Participants

Data were collected from surveys completed by school-going adolescents living in New Caledonia from May 2015 to April 2016. Parents gave informed written consent prior to the children's participation in the study, in line with the legal requirements and the Declaration of Helsinki. The protocol was also approved by the Human Research Ethics Committee of the University of New Caledonia.

**Figure 1:**
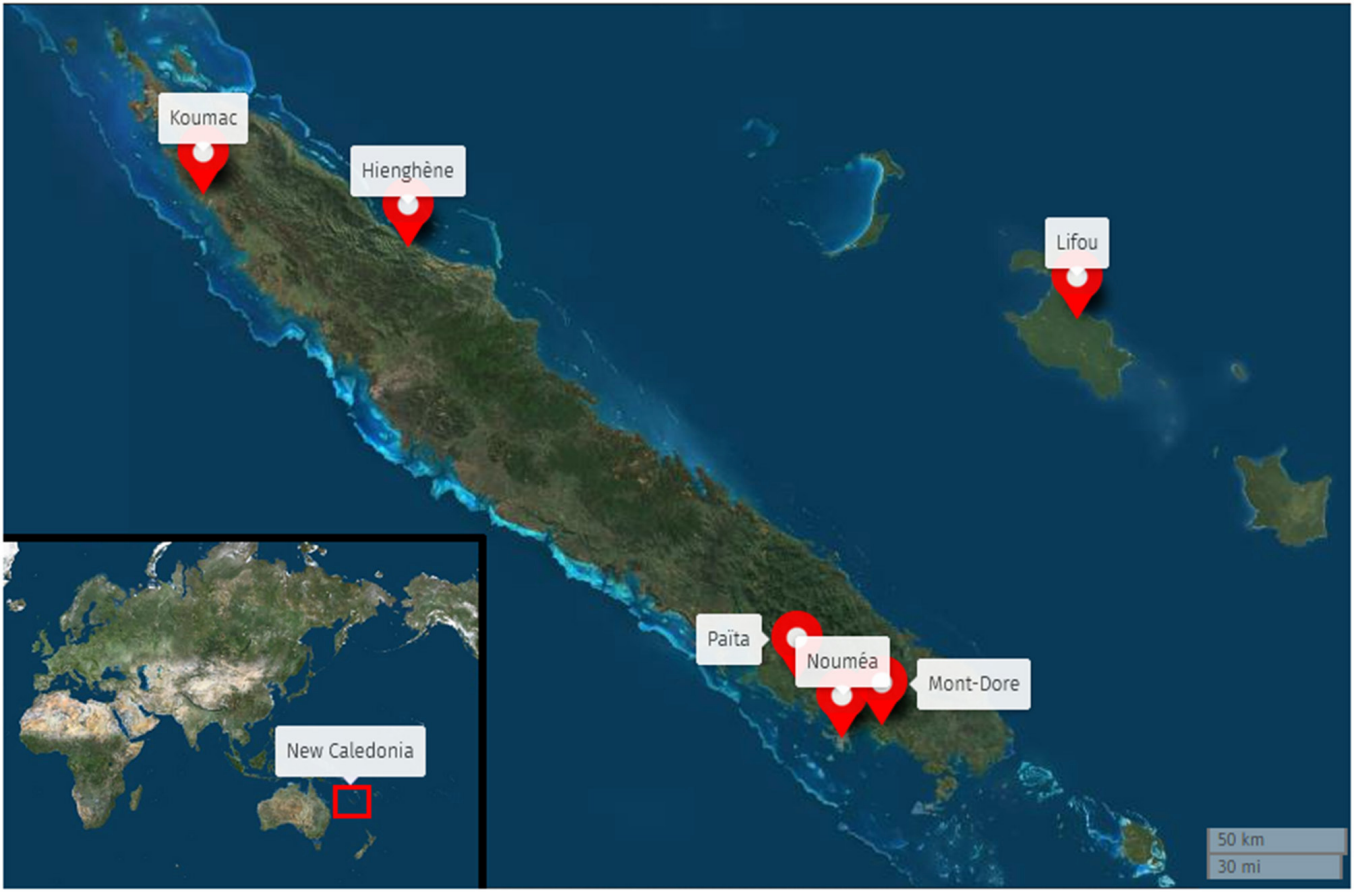
Towns and villages (Koumac, Hienghène, Lifou, Païta, Nouméa, and Mont-Dore) that had schools involved in the study on a New Caledonia map.

New Caledonia is a South Pacific archipelago located between 162–169°E longitude and 19–23°S latitude (Figure 1). It is divided into three provinces (Northern Province, Southern Province, and Loyalty Islands Province) with marked differences in ethnic composition, socioeconomic status (SES), and urbanization. In 2019, the New Caledonia population numbered 271,407. Among them, 75% lived in Southern Province, which includes Grand Noumea (the only urban area on the archipelago) that accounted 67% of the total population. Another 18% lived in Northern Province and 7% in Loyalty Islands Province. This same year, 24% of New Caledonians declared they were Caucasian (European), 41% Melanesian (Kanak), and 10% Polynesian (Tahitian, Wallisian and Futunian), with 24% belonging to other ethnic communities (Indonesian, Ni-Van, Vietnamese, Asiatic, Métis and others). Around 9% of the population was 11–16 years old (51). Thirteen secondary public schools (40%) were located in rural areas and 20 (60%) in urban areas (52).

Five secondary public schools were selected for this study: one in Loyalty Islands Province (rural area), two in Northern Province (east and west coasts, rural areas) and two in Southern Province (Noumea, the capital and only urban area). The selection criterion was school size (*n* > 200) to ensure that sufficient data could be collected in a single visit. Based on this criterion, only one school was eligible in the Loyalty Islands Province, four schools were eligible in the Northern Province (two on each coast), and eight in the Southern Province. Schools were then randomly selected and contacted to obtain agreement from the principal and the teaching team (Figure 1). Two classes were then randomly selected in each of four grades (levels) by a staff member, providing around 180 adolescents per school. In each school, we were able to collect data on 90% of the expected sample. The missing 10% was due to students who either were absent or did not provide parental consent. Adolescents with missing data (*n* = 276) and those from ethnic groups other than Melanesian, Caucasian or Polynesian (*n* = 4) were then excluded because of the small sample size of these groups. Finally, the thresholds of outliers for PA and sedentary time were computed with the following formula:

(1) $$\begin{array}{c} t_{x}=\bar{x}+1.5\times \sigma_{x} \end{array}$$

where *x* is the variable of interest (i.e., PA or sedentary time), *t*_*x*_ the computed threshold, $\bar{x}$ the average of *x*, and σ_*x*_ the standard deviation of *x*. Adolescents with PA > *t*_*PA*_ = 326 min/day or sedentary time > *t*_*ST*_ = 720 min/day were removed from the study (*n* = 55). We note that 18,759 students were enrolled in New Caledonian middle schools in 2015, and this study ultimately included 508 adolescents, representing 2.7% of this population (53).

### Anthropometric Parameters

Anthropometric measurements were made by a trained member of the research team. Adolescents were measured in light indoor clothing with emptied pockets and no shoes. Height was measured with the participant looking straight ahead, heels connecting with a wall mounted stadiometer (Leicester Tanita HR 001, Tanita Corporation, Tokyo, Japan) that was accurate to the nearest 0.1 cm. Weight was determined using a scale (Tanita HA 503, Tanita Corporation, Tokyo, Japan) accurate to the nearest 0.1 kg. Body mass index (BMI) was calculated by dividing weight in kilograms by height squared in meters: BMI = weight/height^2^. The BMI z-scores and percentiles were calculated using the LMS reference values, with the weight status classification defined according to the criteria of the International Obesity Task Force (IOTF) (54), which classifies BMI values according to age and gender as thin (underweight), normal weight, overweight, or obese. The underweight and normal categories were merged, as were the overweight and obese categories, giving a final weight status with only two categories: not overweight (NO) and overweight (O).

### Sociodemographic Characteristics

Demographic data included in the analyses were age, gender, ethnic community and SES. Ethnic community was self-reported by the adolescents using an anonymous questionnaire and categorized as usually processed by the *Institut de la statistique et des études économiques* (ISEE: Institute of Statistics and Economic Studies) census and as recommended by the *Institut national de la santé et de la recherche médicale* (INSERM: the National Institute of Health and Medical Research) report on New Caledonia (55), but they were told they could choose only one ethnic group. The possible ethnic communities were: Kanak (native Melanesian), Caledonian European (European origin and born in New Caledonia), Metropolitan European, Wallisian–Futunian, Tahitian, and other. In the current study, the Caledonian European and Metropolitan European communities were merged into the Caucasian category, and the Wallisian–Futunian and Tahitian communities were merged into the Polynesian category. SES was indexed on the basis of the occupation of the household reference person (defined as the householder with the highest income) using the National Statistics Socio-Economic classification (56). For the present analyses, we generated three categories: managerial and professional occupations (high SES), intermediate occupations (intermediate SES), and routine and manual occupations (low SES).

The 2014 census in New Caledonia (57) used a European standard (58) to assess the degree of urbanization. An urban area was thus defined as a densely populated area comprising at least 50,000 inhabitants in a continuous zone with more than 500 inhabitants per km^2^. A semi-urban area was defined as having more than 50,000 inhabitants in a continuous zone of over 100 inhabitants per km^2^ adjacent to an urban area. A rural area was any area that did not fulfill the conditions required to qualify as being urban or semi-urban.

### Physical Activity and Out-Of-School Sitting Time

Adolescents self-reported their own PA and sitting time *via* the French version of the International Physical Activity Questionnaire–Short Form (IPAQ-SF), which assessed PA over the last 7 days (59–61). The IPAQ-SF classes activity into four categories: sitting, walking, moderate intensity (e.g., leisure cycling), and vigorous intensity (e.g., running or aerobics). Using the IPAQ-SF scoring system, the total number of days and minutes of PA were calculated for each participant as recommended on the IPAQ website (62). The IPAQ is also widely used to provide a proxy of sedentary time (63, 64). We averaged the resulting PA times and out-of-school sitting times in min/day. Then, both the PA and sitting times were dichotomized using the following thresholds: PA time ≥ 60 min/day and out-of-school sitting time ≥ 120 min/day. We focused on out-of-school sitting time because all participants attended school and followed a similar curriculum. We thus considered that sitting time during school hours was similar.

### Socioenvironmental Factors Affecting Physical Activity

The adolescents were asked the following questions about the number of siblings: (1) “How many sisters have you got?” and (2) “How many brothers have you got?” Then the following binary questions (adolescents answered yes or no) were asked in order to assess the impact of the socioenvironmental factors: “When I practice physical activity or sport, it is because…” (3) Peers: “… a friend comes with me” [Yes/No]; (4) Family: “… a family member comes with me” [Yes/No]; (5) Safety: “…I feel safe” [Yes/No]; and (6) Accessibility: “… it is easy to find a place where I can be active” [Yes/No]. Questions were extracted from the study of Jego et al. (65) and adapted to the New Caledonian context.

### Statistics

The analysis consisted of assessing differences in PA, sitting time, social factors associated with PA or sport, and anthropometric variables (mass, height, BMI, IOTF z-score, and weight status) according to sex, ethnic community and place of living (rural or urban). All the results are presented for female and male adolescents.

The differences between two groups (e.g., rural and urban) were determined with a means equality test (Student or Welch *t*-test) for continuous variables. When conditions for the application of the *t*-test were not verified, the non-parametric Wilcoxon test was used. The differences according to a factor having more than two categories (ethnic community: Melanesian, Caucasian, and Polynesian) were tested using a one-way ANOVA when conditions of normality (or at least a distribution close to the normal distribution and a sufficient sample size) and homoscedasticity were satisfied, otherwise the Kruskal-Wallis test was implemented. *Post-hoc* tests were implemented when the differences between factor groups were significant after the one-way ANOVA or Kruskal-Wallis test: the Tukey *post-hoc* test when using a one-way ANOVA and the Steel-Dwass-Critchlow-Fligner multiple comparison test when using the Kruskal-Wallis test (66). For significant differences in categorical variables, we used the χ^2^ test when the Cochran rules were not violated, otherwise the Fisher exact test was implemented. When factors had more than two categories, the *post-hoc* test *p*-values were corrected with the Bonferroni adjustment in cases of significance. Indicators for effect size (EFI) were computed and then categorized in effect size magnitudes. EFI for numerical factors were *η*^2^ and Kruskal-Wallis effect size and EFI for categorical factors was Cramer V. The effect size magnitude for numerical factors was determined as follows: small (S: EFI <0.06), moderate (M: 0.06 ≤ EFI < 0.14) and large (L: EFI ≥ 0.14). The effect size magnitude for categorical factors was determined as follows: small (S: EFI <0.21 when df = 2 and EFI < 0.15 when df = 4), moderate (M: 0.21 ≤ EFI < 0.35 when df = 2 and 0.15 ≤ EFI < 0.25 when df = 4), and large (L: EFI ≥ 0.35 when df = 2 and EFI ≥ 0.25 when df = 4) (67).

Factors associated with active time (PA ≥ 60 min/day) and sitting time (out-of-school sitting time ≥ 120 min/day) were first assessed *via* odds ratios (OR) and odds ratios adjusted for socioeconomic confounding factors and interaction factors (OR_adj_). Finally, multivariate logistic regressions were computed in order to assess the association between the IOTF weight status (with two categories: NO and O) and active vs. sitting time.

We determined the inclusion of the confounding factors and interaction factors (Tables 1–3) in the adjusted models (for OR_adj_ computing) by a background selection. Only confounding factors and interaction factors whose *p*-values were lower than 0.20 (*p* < 0.20) were included in the models.

**Table 1:** Associations between PA time and socioenvironmental factors one by one adjusted with the confounding factors (place of living, ethnic community, age) and interactions.

**PA time ≥ 60 min/day**	**Female**				**Male**			
	**OR [95% CI]**	***p*-value**	**OR_**adj**_ [95% CI]**	***p*-value**	**OR [95% CI]**	***p*-value**	**OR_**adj**_ [95% CI]**	***p*-value**
Siblings	1.08 [0.97;1.22]	0.159	1.01 [0.90;1.14]	0.868^C^^,^^A^	1.17 [1.00;1.38]	0.061	1.27 [1.05;1.59]	0.025^L^^,^^#1^
Peers	1.74 [1.03;2.94]	0.039	1.79 [1.02;3.14]	0.041^C^^,^^A^	1.48 [0.83;2.64]	0.186	3.05 [1.09;8.89]	0.036^C^^,^^#1^
Family	1.67 [0.98;2.84]	0.056	2.35 [1.22;4.52]	0.010^L^^,^^C^^,^^A^^,^^*^	1.54 [0.87;2.74]	0.139	1.54 [0.87;2.74]	0.139
Safety	2.87 [1.69;4.91]	<0.001	2.85 [1.63;5.01]	<0.001^C^^,^^A^	4.14 [2.28;7.64]	<0.001	4.45 [2.42;8.36]	<0.001^L^
Accessibility	1.21 [0.67;2.12]	0.520	1.53 [0.82;2.82]	0.179^C^^,^^A^	2.82 [1.48;5.39]	0.002	2.94 [1.51;5.77]	0.002^C^

*OR, odds-ratio; OR_adj_, adjusted odds-ratio; CI, confidence interval*.

*Confounding factors included in the model*.

L * Place of living*.

C * Ethnic community*.

S * SES*.

A * Age*.

*Interaction factors included in the model*.

* * Family with Place of living (OR_adj,Urban_ = 0.22, p_Urban_ = 0.024)*.

#1 * Siblings with Place of living (OR_adj,Urban_ = 0.60, p_Urban_ = 0.018)*.

#2 * Peers with Ethnic community (OR_adj,Melanesian_ = 0.38, p_Melanesian_ = 0.147, OR_adj,Polynesian_ = 0.16, p_Polynesian_ = 0.131)*.

**Table 2:** Associations between out-of-school sitting time and socioenvironmental factors one by one adjusted with the confounding factors (place of living, ethnic community, age) and interactions.

**Out-of-school sitting time ≥ 120 min/day**	**Female**				**Male**			
	**OR [95% CI]**	***p*-value**	**OR_**adj**_ [95% CI]**	***p*-value**	**OR [95% CI]**	***p*-value**	**OR_**adj**_ [95% CI]**	***p*-value**
Siblings	0.87 [0.78;0.96]	0.007	0.93 [0.83;1.03]	0.179^C^^,^^S^	0.91 [0.80;1.03]	0.160	0.62 [0.39;0.90]	0.019^C^^,^^A^^,^^#1^
Peers	0.90 [0.56;1.46]	0.673	0.20 [0.04;0.79]	0.031^C^^,^^S^^,^^*1^	0.83 [0.48;1.43]	0.515	0.82 [0.46;1.44]	0.482^C^
Family	0.90 [0.55;1.46]	0.658	0.95 [0.56;1.60]	0.834^C^^,^^S^	0.88 [0.52;1.48]	0.622	0.82 [0.43;1.58]	0.558^L^^,^^C^^,^^A^^,^^#2^
Safety	1.51 [0.93;2.45]	0.098	0.75 [0.25;2.17]	0.595^L^^,^^C^^,^^S^^,^^*2^	0.67 [0.38;1.18]	0.170	0.00 [0.00;0.52]	0.030^C^^,^^A^^,^^*3^
Accessibility	1.61 [0.95;2.75]	0.080	0.01 [0.00;2.81]	0.111^C^^,^^S^^,^^A^^,^^*3^	0.95 [0.51;1.78]	0.882	0.86 [0.45;1.74]	0.7341^C^^,^^A^

*OR, odds-ratio; OR_adj_, adjusted odds-ratio; CI, confidence interval*.

*Confounding factors included in the model*.

L * Place of living*.

C * Ethnic community*.

S * SES*.

A * Age*.

*Interaction factors included in the model*.

*1 * Peers with Ethnic community (OR_adj,Melanesian_ = 1.65, p_Melanesian_ = 0.424; OR_adj,Polynesian_ = 0.15, p_Polynesian_ = 0.197), Peers with SES (OR_adj,Intermediate_ = 5.12, p_Intermediate_ = 0.045; OR_adj,Low_ = 4.52, p_Low_ = 0.043)*.

*2 * Safety with Place of living (OR_adj,Urban_ = 0.32, p_Urban_ = 0.144), Safety with Ethnic community (OR_adj,Melanesian_ = 5.18, p_Melanesian_ = 0.009; OR_adj,Polynesian_ = 3.91, p_Polynesian_ = 0.317)*.

*3 * Accessibility with Age (OR_adj_ = 1.45, p = 0.085)*.

#1 * Siblings with Ethnic community (OR_adj,Melanesian_ = 1.65, p_Melanesian_ = 0.021; OR_adj,Polynesian_ = 2.63, p_Polynesian_ = 0.093)*.

#2 * Family with Place of living (OR_adj,Urban_ = 2.75, p_Urban_ = 0.144)*.

#3 * Safety with Age (OR_adj_ = 1.60, p = 0.034)*.

**Table 3:** Association between PA and out-of-school sitting time with weight status (being overweight) in female and male adolescents.

**IOTF weight status**	**Female**				**Male**			
	**OR [95% CI]**	***p*** **-value**	**OR**_**adj**_ **[95% CI]** ^**AF**^	***p*** **-value**	**OR [95% CI]**	***p*** **-value**	**OR**_**adj**_ **[95% CI]** ^**AM**^	***p*** **-value**
Active time ≥ 60 min/day	0.79 [0.47;1.34]	0.376	0.62 [0.34;1.10]	0.103	0.84 [0.47;1.54]	0.061	0.28 [0.08;0.95]	0.044
Out-of-school sitting time ≥ 120 min/day	1.18 [0.73;1.91]	0.501	1.55 [0.92;2.62]	0.101	0.41 [0.23;0.71]	0.002	0.39 [0.20;0.73]	0.004

*OR, odds-ratio; OR_adj_, adjusted odds-ratio; CI, confidence interval*.

AF *Adjusted with Place of living (OR_adj,Urban_ = 0.28, p_Urban_ = 0.002) and Ethnic community (OR_adj,Melanesian_ = 1.99, p_Melanesian_ = 0.030, OR_adj,Polynesian_ = 7.86, p_Polynesian_ = 0.001)*.

AM *Adjusted with Ethnic community (OR_adj,Melanesian_ = 0.68, p_Melanesian_ = 0.520, OR_adj,Polynesian_ = 5.16, p_Polynesian_ = 0.092), SES (OR_adj,Intermediate_ = 2.49, p_Intermediate_ = 0.028, OR_adj,Low_ = 2.20, p_Low_ = 0.046) and interactions between Ethnic community and Active time (OR_adj,Melanesian_ = 3.90, p_Melanesian_ = 0.074, OR_adj,Polynesian_ = 0.87, p_Polynesian_ = 0.911)*.

The analyses were conducted using R 3.5.1 (68) with a first species risk probability level set at *α* = 0.05.

## Results

### Physical Activity and Out-of-School Sitting Time

Overall, around 66% of the adolescents, both females and males, declared engaging in PA more than 60 min/day. Moreover, our results showed that Melanesian adolescents living in rural areas were the most physically active and spent the least out-of-school sitting time. This active living was not found in the urban environment.

**Figure 2:**
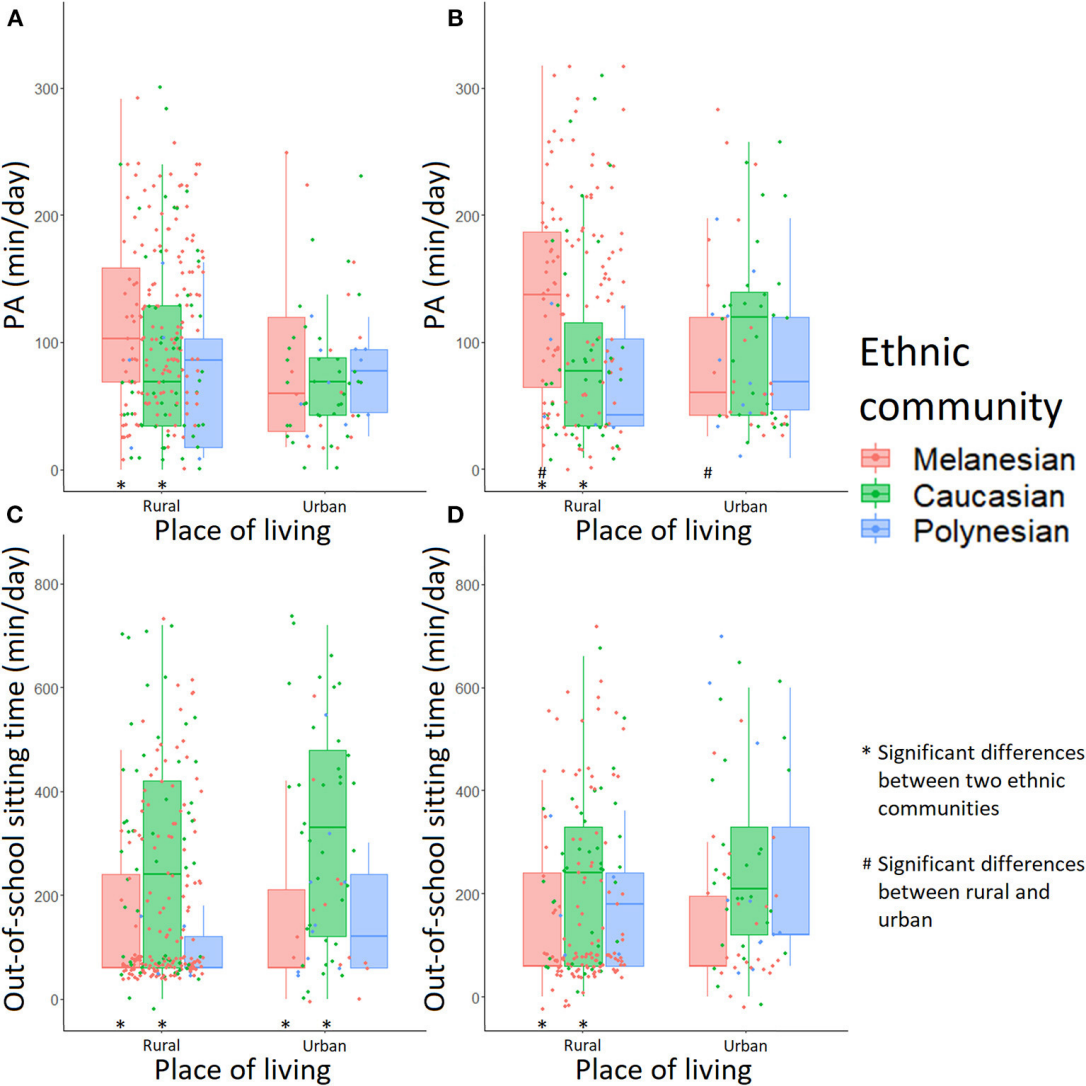
Time spent in physical activity and sitting time in adolescents according to ethnic community and place of living: **(A)** PA in female adolescents, **(B)** PA in male adolescents, **(C)** out-of-school sitting time in female adolescents and **(D)** out-of-school sitting time in male adolescents. A “*” note means a significant difference between two ethnic communities in a single place of living; a “#” note means a significant difference between rural and urban environment in a single ethnic community.

A greater proportion of Melanesian adolescents engaged in 60 min/day of PA in rural compared to urban areas (75.78% vs. 46.67% in girls, *p* = 0.028; 74.79% vs. 45.83% in boys, *p* = 0.010; Supplementary Tables 1, 2). Figure 2 shows that male Melanesian adolescents who lived in a rural environment spent on average 133 min/day in PA compared to 95 min/day for those living in an urban environment (*p* = 0.028; Supplementary Table 2). While a similar trend was observed for female adolescents (115 min/day in rural vs. 88 min/day in urban, *p* = 0.118), this was not statistically significant.

In the rural environment, both female and male Melanesian adolescents were more physically active compared with Caucasians (115 vs. 93 min/day in girls, *p* = 0.018; 133 vs. 97 min/day in boys, *p* = 0.018; Figure 2, Supplementary Tables 3, 4). In this environment, 75.78% of Melanesian vs. 52.73% of Caucasian female adolescents engaged in PA for at least 60 min/day on average (*p* = 0.006; Supplementary Table 3). Both male and female Melanesian adolescents spent less out-of-school sitting time on average than their Caucasian counterparts in the rural environment (166 vs. 271 min/day in girls, *p* = 0.008; 170 vs. 231 min/day in boys, *p* = 0.019; Figure 2, Supplementary Tables 3, 4), whereas this was true only for females in the urban environment (156 vs. 332 min/day, *p* = 0.019; Figure 2, Supplementary Table 3). In rural areas, 42.86% of Melanesian vs. 71.79% of Caucasian male adolescents declared staying seated more than 120 min/day out of school (*p* = 0.009; Supplementary Tables 2, 4).

### Factors Associated With PA and Out-Of-School Sitting Time

**Figure 3:**
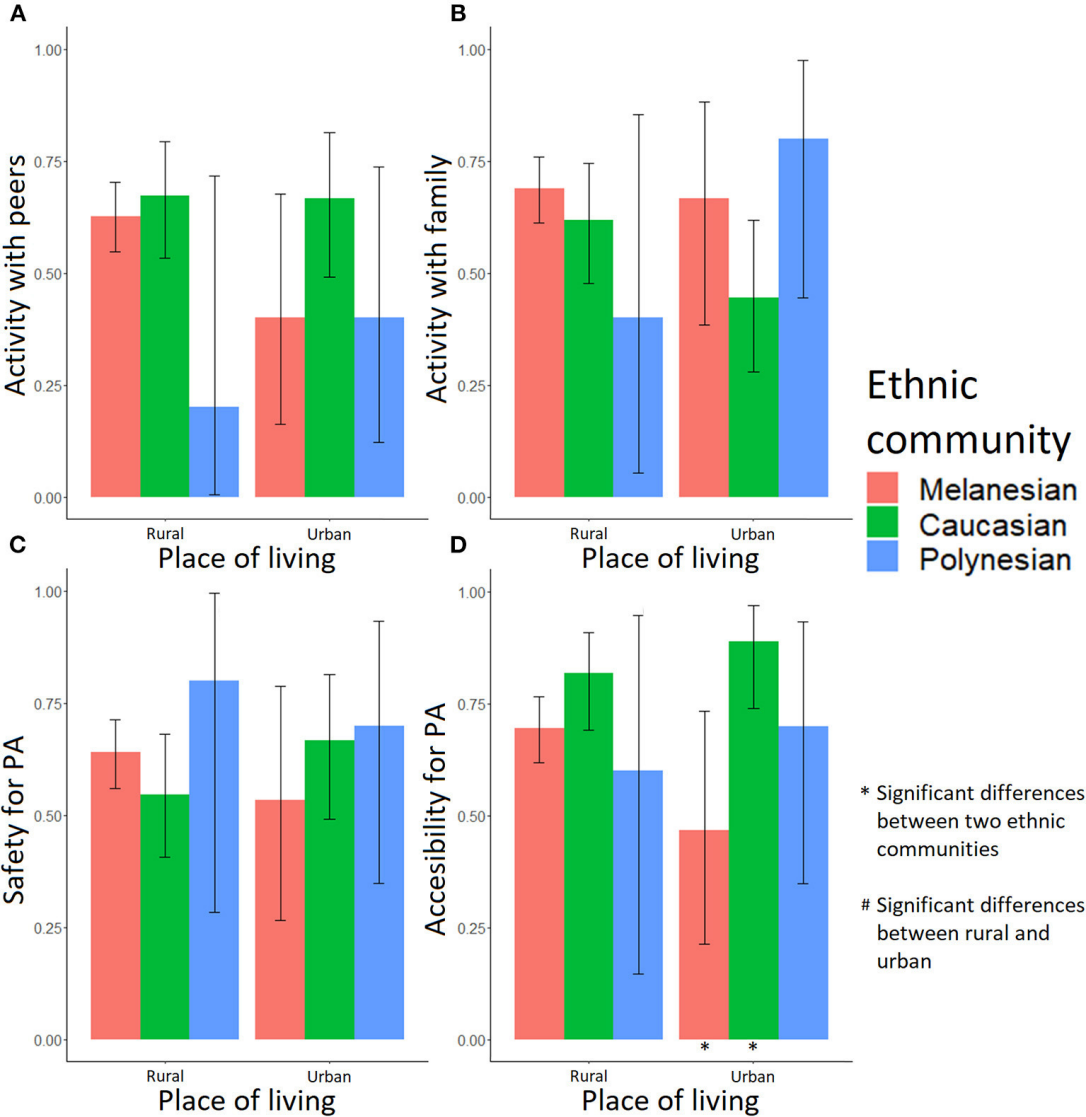
Fractions of female adolescents for whom the socioenvironmental factors are important in PA practice. Activity: **(A)** with peers, **(B)** with family, **(C)** in safety, and **(D)** easy access. A “*” note means a significant difference between two ethnic communities in a single place of living; a “#” note means a significant difference between rural and urban environment in a single ethnic community.

**Figure 4:**
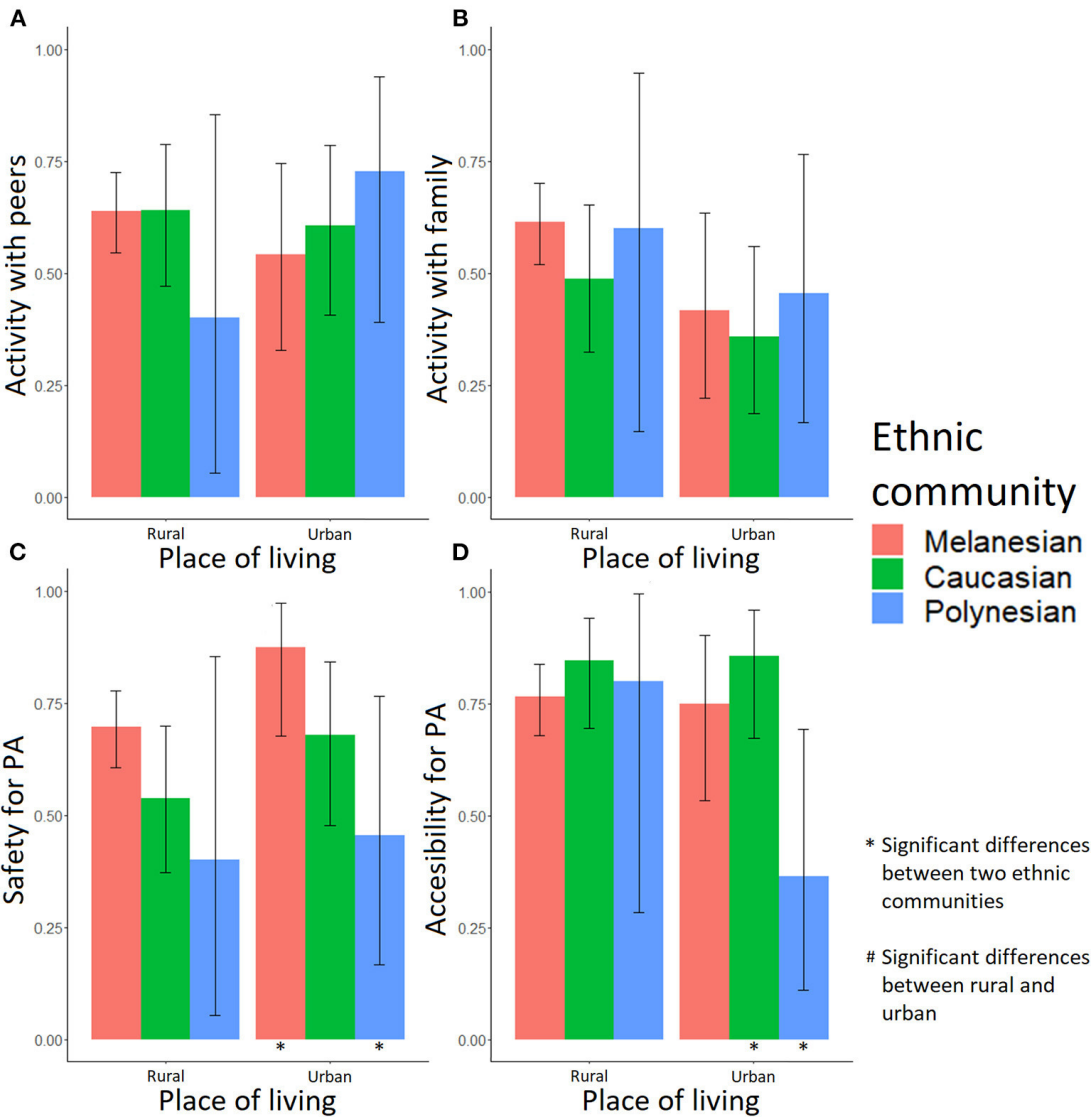
Fractions of male adolescents for whom the socioenvironmental factors are important in PA practice. Activity: **(A)** with peers, **(B)** with family, **(C)** in safety, and **(D)** easy access. A “*” note means a significant difference between two ethnic communities in a single place of living; a “#” note means a significant difference between rural and urban environment in a single ethnic community.

Most adolescents identified specific contexts (peers, family, safety and accessibility) as important (answered yes to the binary questions) when it comes to engaging in PA (Supplementary Tables 1, 2, Figures 3, 4). Accessibility was especially important for both Melanesian and Caucasian adolescents (68% yes in Melanesian females, 76% yes in Melanesian males, and 85% yes in Caucasians), while safety was more consistently reported as important by Melanesian adolescents (63% yes in females and 73% yes in males). In urban areas, accessibility to a suitable place was identified as important more consistently by female Caucasian (88.89%) than Melanesian (46.67%) adolescents (*p* = 0.008; Figure 3D, Supplementary Table 3). An accessible area for PA was also an important factor for both males (76.22% Melanesians, 85.07% Caucasians, and 50.00% Polynesians; Supplementary Table 2) and females (67.61% Melanesians, 84.62% Caucasians, and 66.67% Polynesians; Supplementary Table 1). For male adolescents, having access to a safe area was also important (72.73% Melanesians, 59.70% Caucasians, and 43.75% Polynesians; Figure 4C, Supplementary Table 2) and this was particularly true for Melanesians living in the urban area compared with Polynesians (87.50% vs. 45.45%, *p* = 0.044; Supplementary Table 4).

**Figure 5:**
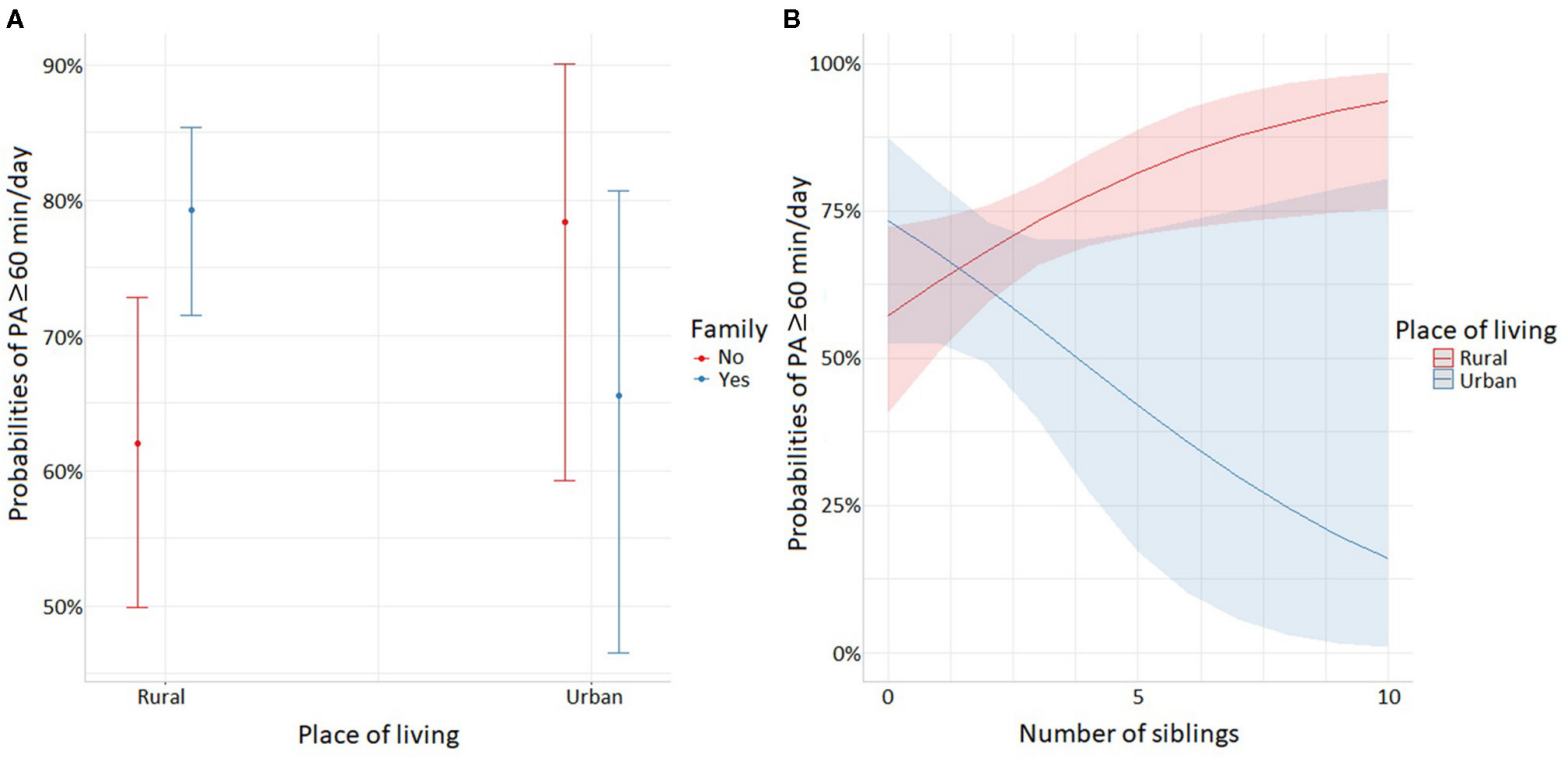
Significant interactions for PA time: **(A)** interactions between place of living and family in female adolescents, **(B)** interactions between place of living and siblings in male adolescents.

Some of these socioenvironmental factors appeared associated with PA and out-of-school sitting time. In female adolescents, practicing with peers (OR_adj_ = 1.79, *p* = 0.041), with family (OR_adj_ = 2.35, *p* = 0.010) and in a safe environment (OR_adj_ = 2.85, *p* < 0.001) were positively associated with PA (Table 1). However, interactions between place of living and family should be taken into account. As shown in Figure 5A, the effect of engaging with family members was positive on PA in girls living in rural areas, while in urban areas the effect was not significant. In male adolescents, the number of siblings (OR_adj_ = 1.27, *p* = 0.025), peers (OR_adj_ = 3.05, *p* = 0.036), safety (OR_adj_ = 4.45, *p* < 0.001), and accessibility (OR_adj_ = 2.94, *p* = 0.002) were positively associated with PA (Table 1). There were significant interactions between place of living and siblings (OR_adj,Urban_ = 0.60, p_Urban_ = 0.018). Figure 5B highlights that the impact of siblings seemed positive, especially in rural areas, when male adolescents had more than three brothers and sisters.

**Figure 6:**
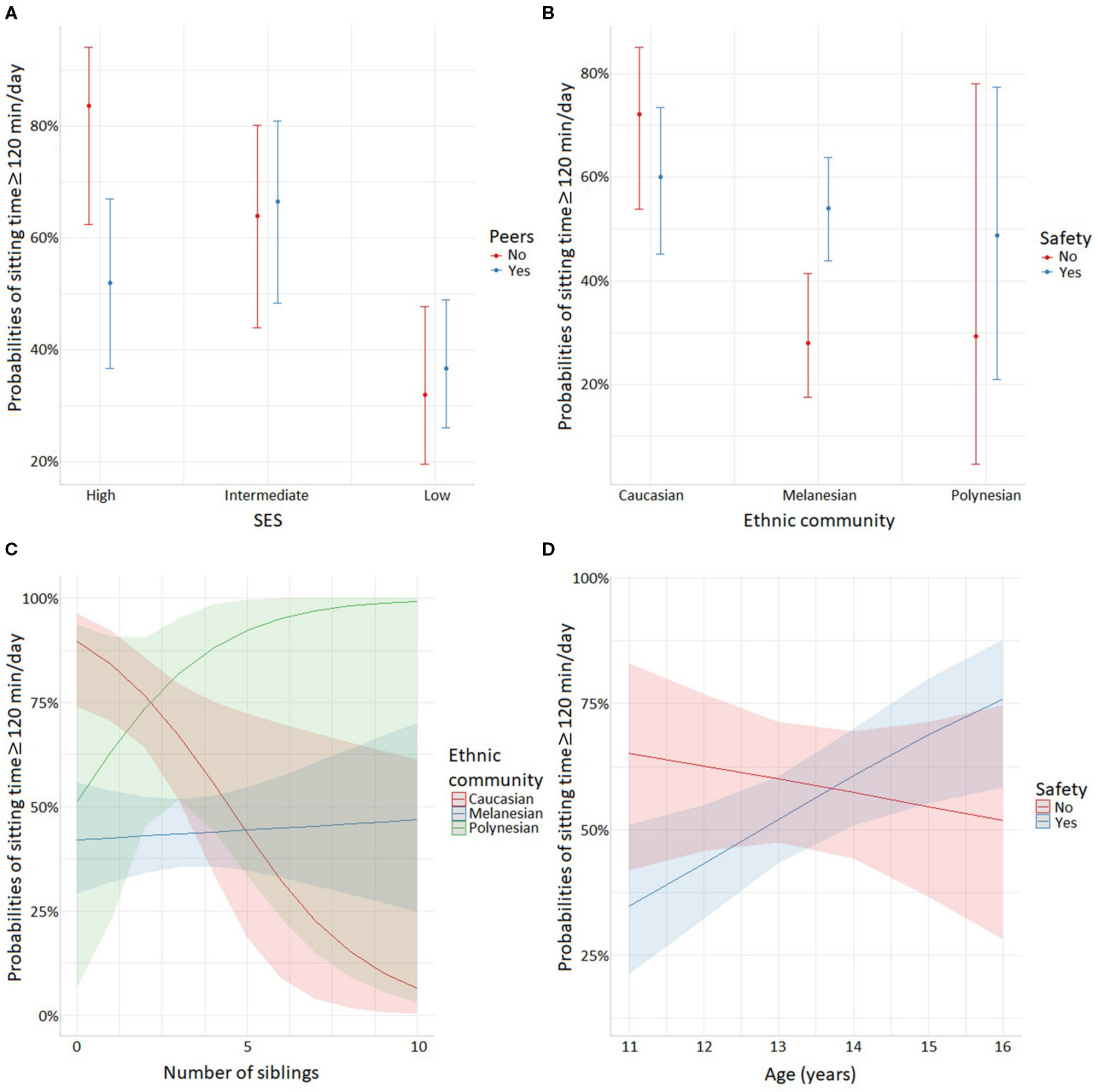
Significant interactions for out-of-school sitting time: **(A)** interactions between SES and importance of practicing PA with peers in female adolescents, **(B)** interactions between ethnic community and safety in female adolescents, **(C)** interactions between ethnic community and siblings in male adolescents, **(D)** interactions between age and safety in male adolescents.

Only the factor peers was negatively associated with the out-of-school sitting time of female adolescents (OR_adj_ = 0.20, *p* = 0.031, Table 2). There was a significant interaction between SES and peers (OR_adj,Intermediate_ = 5.12, p_Intermediate_ = 0.045; OR_adj,Low_ = 4.52, p_Low_ = 0.043), and between ethnic community and safety (OR_adj,Melanesian_ = 5.18, p_Melanesian_ = 0.009). As shown in Figure 6A, peers had a negative effect on sitting time especially in high SES. Figure 6B shows that female Melanesian adolescents claiming that feeling safe when practicing PA is important (safety) were more likely to declare a sitting time ≥ 120 min/day when compared with female Melanesian adolescents for whom safety was not so important. In male adolescents, siblings (OR_adj_ = 0.62, *p* = 0.019) and safety (OR_adj_ = 0.00, *p* = 0.030) were negatively associated with out-of-school sitting time. We found significant interactions between ethnic community and siblings (OR_adj,Melanesian_ = 1.65, p_Melanesian_ = 0.021; OR_adj,Polynesian_ = 2.63, p_Polynesian_ = 0.093) and between age and safety (OR_adj_ = 1.60, *p* = 0.034). Figure 6C shows that the number of siblings had a negative association with sitting time only in Caucasians, whereas it was positive in Polynesian male adolescents. Moreover, safety seemed negatively associated with sitting time in male adolescents younger than 13 years old (Figure 6D).

### Anthropometry and Sociodemographic Factors

Around 40% of the Melanesian, <25% of the Caucasian, and 60% of the Polynesian adolescents were O (Supplementary Tables 1, 2), but differences were observed according to sex and place of living. The IOTF z-score average in Melanesian female adolescents living rural areas was 1.03, whereas it was 0.24 in those living in urban areas (*p* = 0.006; Supplementary Table 1). Caucasian female adolescents had a higher IOTF z-score in rural areas than in urban areas (0.89 in rural vs. 0.02 in urban, *p* = 0.001), and the prevalence of O was higher in rural areas than in urban areas (34.55% O in rural vs. 11.11% O in urban, *p* = 0.023). In Caucasian male adolescents, the IOTF z-score was higher in rural areas than in urban areas (0.64 vs. −0.02, *p* = 0.031; Supplementary Table 2).

Supplementary Table 3 shows no significant anthropometric differences in rural female adolescents between ethnic communities. In urban areas, there were significant differences for the IOTF z-score (0.02 vs. 1.11, *p* = 0.017) and IOTF weight status (11.11% O vs. 50.00% O, *p* = 0.045) when Caucasians and Polynesians were compared. Supplementary Table 4 shows significant differences in male adolescents between ethnic communities, but these differences were not systematically found in comparisons according to the place of living. There were no differences in rural areas according to ethnic community. In urban areas, differences were significant for the IOTF z-score (0.96 in Melanesians vs. −0.02 in Caucasians, *p* = 0.023; −0.02 in Caucasians vs. 1.16 in Polynesians, *p* = 0.035) and IOTF weight status (*p* = 0.024 but there was no significance with the *post-hoc* test).

### Association Between PA, Out-of-School Sitting Time and Weight Status

Through adjusted odds ratios, Table 3 shows that the weight status associated with being active (i.e., PA ≥ 60 min/day) and with out-of-school sitting time ≥ 120 min/day was controlled by socioeconomic factors. In females, place of living and ethnic community were associated with IOTF weight status, whereas PA and sitting time were not. A female adolescent living in the urban context was less likely to be overweight or obese (OR_adj_ = 0.28, *p* = 0.002) but Melanesian (OR_adj_ = 1.99, *p* = 0.030) and Polynesian (OR_adj_ = 7.86, *p* = 0.001) females were more likely to be overweight or obese than the Caucasian females. In male adolescents, SES and both PA and sitting time were associated with IOTF weight status. Boys from intermediate (OR_adj_ = 2.49, *p* = 0.028) or low (OR_adj_ = 2.20, *p* = 0.046) SES were more likely to be overweight or obese than boys from high SES. Active male adolescents (PA ≥ 60 min/day) and those who declared sitting ≥ 120 min/day out of school were less likely to be overweight or obese (OR_adj_ = 0.28, *p* = 0.044 and OR_adj_ = 0.39, *p* = 0.004, respectively).

**Figure 7:**
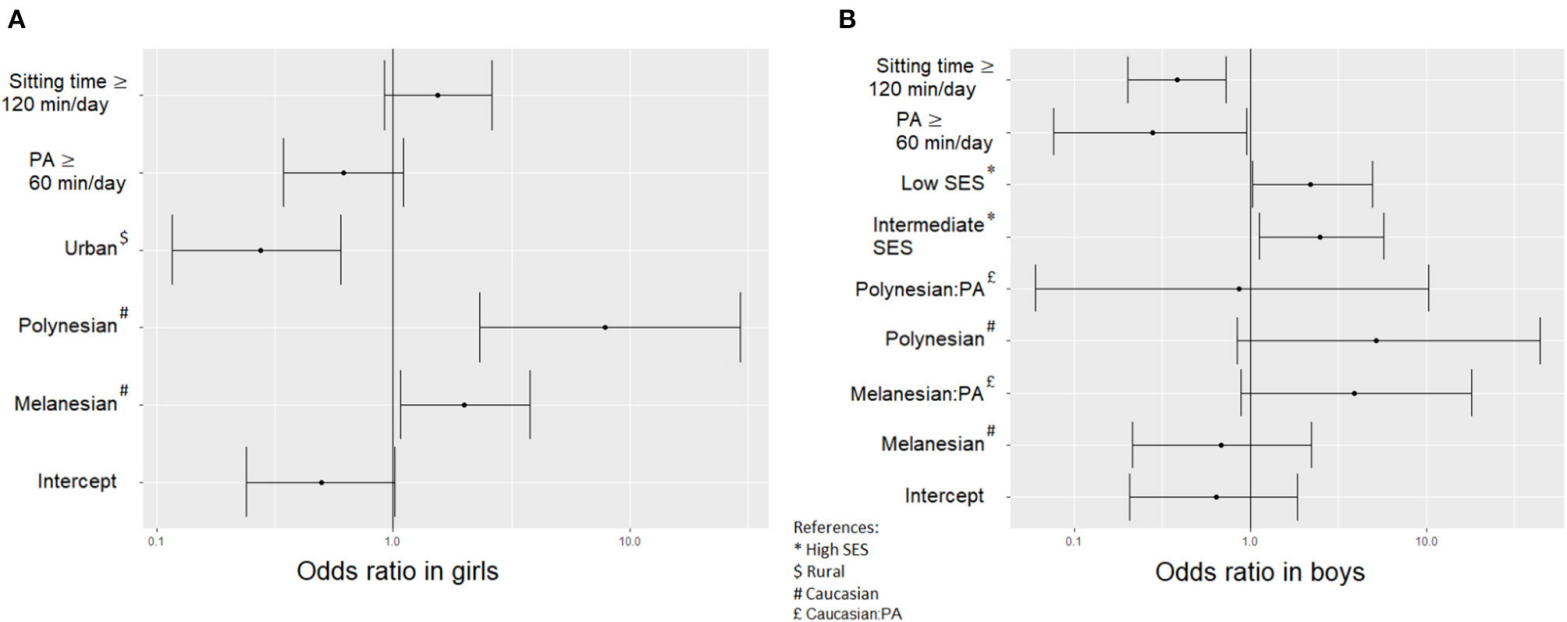
Odds ratio and error bars for the multivariate logistic regressions estimating the chance of being overweight or obese in **(A)** female adolescents and **(B)** male adolescents.

Based on the above-described results, Figure 7 shows the strength of association between the socioeconomic and PA factors on the one hand and the overweight and obesity risk on the other hand.

## Discussion

This study, which focused on PA in a pluri-ethnic adolescent population, showed that 66% of adolescents reported 60 min or more of daily PA with disparities driven by place of living and ethnic community. More specifically, Melanesian adolescents were generally more active and spent less sitting time than Caucasians. Melanesian adolescent boys living in rural areas presented the most active lifestyle.

Belonging to an ethnic community or SES was associated with PA and sitting time in girls but not in boys. In addition, feeling safe was a strong driver for PA in both male and female adolescents.

Finally, being active (PA ≥ 60 min/day) and out-of-school sitting time ≥ 120 min/day were not significant predictors for weight status in female adolescents, while they seemed to prevent overweight in adolescents boys.

### Physical Activity

The proportion of adolescents who regularly engaged in PA in New Caledonia (66%) is fairly encouraging, when compared with results of previous studies in other Pacific Island Countries and Territories (PICTs) (10, 34). Indeed, in a previous study on adolescents 13–17 years old, Kessaram et al. (34) found that 17.5% (in Nauru) to 45.7% (in Vanuatu) of males and 12.5% (in Nauru) to 46.7% (in Vanuatu) of females were physically active, e.g., engaged in 60 min/day of PA, as assessed by the Global School-Based Student Health Survey 2011 (34). However, the more restrictive definition of an “active person” in their study, which was different from ours (at least 60 min/day of PA for at least 5 days in Kessaram et al. and at least 60 min/day on average in the current study), could explain this difference.

In this study, we did not find significant differences for the average amounts of PA in male and female adolescents except in rural Melanesians (see Supplementary Table 1). This is an unprecedented result in New Caledonia that contrasts with previous studies in which authors found significant differences according to gender (19, 23, 34, 69, 70).

More Melanesian adolescents reached 60 min/day of PA in rural compared to urban areas, both for girls (75.78% in rural and 46.67% in urban) and boys (74.79% in rural and 45.83% in urban). A similar association was found in populations of Turkey and the Republic of Cameroon (71, 72). Because of the industrialized urban life, schoolchildren tend to spend much more time in sedentary activities such as reading, playing video games or watching TV. Indeed, they have less chance to play outside when compared with their rural counterparts (72). In contrast, there was an inverse association in Polish adolescents between place of residence and PA, with a higher percentage of adolescents with low PA in a rural environment (48). The authors explained that the lack of PA time in the rural environment may have been due to a high dependence on driving. In our study, the higher PA level in Melanesian adolescents living in rural areas may be explained by the strong participation in community life, especially in tribes. Indeed, most of the Melanesians living in rural areas have kept a tribal lifestyle, which implies engaging in a number of physical activities such as hunting, fishing, and cultivating fields (30, 73).

Ethnicity was associated with PA and sedentary time in New Caledonia, which is in line with previous studies conducted in other countries (2, 8, 23, 49). In a rural environment, Melanesian adolescents, both females and males, were significantly more active (higher PA time and lower out-of-school sitting time) than Caucasians. Caucasians living in rural areas, as well as Melanesians, were likely to engage in activities such as hunting and fishing. However, as highlighted before, the community lifestyle followed by Melanesians may require more PA time to meet the community needs and may also provide more opportunities for pleasurable PA when extended family and friends gather for leisure time (30, 73). Moreover, practicing PA with peers has been identified by adolescents as an important component for PA participation, as discussed further below (16).

The socioeconomic and socioenvironmental factors related to PA were different according to gender in New Caledonia. In adolescent girls, ethnic community, age, peers, family and feeling safe were associated with PA ≥ 60 min/day. In adolescent boys, PA was related to the number of siblings, peers, feeling safe and accessibility. In addition, while engaging in PA with peers was negatively associated with a high sitting time (out-of-school sitting time ≥ 120 min/day) for female adolescents, siblings and safety were negatively associated with a high sitting time in male adolescents. Several studies have reported that general social support is determinant for adolescent engagement in PA (8, 18, 23, 49). However, the current study suggests that friendship had more impact on adolescent girls' sitting time than family ties. A study conducted in Hawaiian adolescents revealed that they were more likely to spend sedentary time with parents and active time with friends (21). Indeed, during childhood and the preadolescent years, parental modeling of PA plays a crucial role in lifestyle behavior and establishing a social norm (74). As children mature, they become more independent and the association between parental and children's lifestyles start to diverge (22). For this reason, studies have reported inconsistent associations between PA and parental behavior, modeling, and encouragement, depending on the children's age (22, 23). Other studies have pointed to the importance of friendship for engaging in PA (16, 26), particularly because it expands the number and types of PA that adolescents can participate in Rittenhouse et al. (75) and Salvy and Bowker (16). Also, with regard to our finding that family ties were associated with PA in girls but not in boys, we should take into account that the place of women is central in household activities in Pacific communities (76), whereas men carry out their customary activities outside the household, i.e., with friends and extended family members (45–47).

Although feeling safe has not always been found to be an important factor in previous studies (8, 15), it was an important driver of PA in this study independently of the place of residence or ethnicity. Our survey does not allow us to further explore the reasons behind the feeling of safety expressed by the adolescents. Did they have in mind the risk of injury, incivilities, or aggressions or the risk related to traffic? They may have been thinking about one or more of these risks or another kind of risk; this study did not provide a straight answer to this question.

Easy access to a place perceived as suitable for engaging in PA or sports was positively associated with PA in boys but not in girls. The question about accessibility was designed to elicit information about the proximity to infrastructures that would facilitate adolescent engagement, especially in PA and sports. In New Caledonia, adolescents living in the urban environment can easily access sports fields. Although most villages and tribes (in rural environments) have such infrastructures, they provide less diversity. Yet, interestingly, even though some of the rural adolescents may have lived quite far from PA infrastructures, this did not seem to limit their engagement in PA, probably because the rural community context offered other opportunities, such as open areas, beaches or marine activities. Other adolescents have reported an inconsistent relationship between PA and access to parks, recreation facilities and street connectivity (15). In the urban environment, there were probably some barriers (including safety and appropriate infrastructures) since PA was lower. Environmental and policy modifications, such as improving infrastructure and safety for walking, have been suggested as effective ways to address these barriers to PA (8, 15). Overall, WHO suggested international best practices for implementing PA programs in developing countries that could help populations increase the daily PA levels (14, 45).

### PA, Sitting-Time, and Weight Status

In this study, about 38% of the girls and 34% of the boys were overweight or obese (being O). The analysis of the predictors of being O revealed that both PA and sitting time were not significantly predictive in girls, whereas they were in boys. Previous studies found that large amounts of sedentary behavior but not PA were associated with overweight and obesity (8, 77, 78). The PA in our study was not necessarily MVPA, and therefore the adolescents reaching 60 min/day of PA did not necessarily fulfill the international recommendations of 60 min/day of MVPA. It may be that on average the intensity of PA was light to moderate rather than moderate to vigorous. It may also be that the girls engaged in different types of PA compared to boys. One surprising result is that larger amounts of sitting time were not a predictor of overweight in the adolescent boys. One explanation may be that the boys engaged in more intense PA and that sitting time was more quiet time to rest. Indeed, the relationship between sitting and overweight is largely determined by gaming and recreational screen time. It is important to note that adolescents living far from school have to use public transport to go to school and then to come back home, and this too implies a fairly long sitting time.

Nevertheless, the association between PA and weight status, especially overweight and obesity, is complex. Bauman et al. (8) highlighted that the association between overweight or obesity and PA can go in both directions. While it is assumed that PA can help with weight loss in overweight individuals, some studies have challenged this assumption, pointing out that insufficient PA may lead to obesity but that obesity can be a driver of physical inactivity (8).

A study conducted in Vanuatu found negative associations between physical activities such as gardening and housekeeping and indicators of weight status including BMI, body fat mass percentage, waist circumference and the sum of skinfolds (31). The associations between sedentary activities (TV, radio, and video) and indicators of weight status were also negative, which is consistent with our study. The authors highlighted the finding that PA was high even in the individuals who spent a lot of time in sedentary recreational activities. The study also investigated nutrition and found a positive association between obesity and consumption of non-traditional food, consistent with another study (73). A recent study including adolescents living in New Caledonia revealed that a large number of adolescents regularly consume sugar-sweetened beverages (79). The authors did not find a significant association between consumption of these beverages and weight status or PA. Although consumption of sugar-sweetened beverages or other unhealthy food products like snacks and fast foods is generally associated with sedentary time, including uninterrupted sitting times or long periods screen watching (4, 13), it might also be associated with time spent with friends or even with active time (80, 81). When adolescents spend time together, they do not necessarily sit but they could share unhealthy food and beverages (82, 83). Thus, they might consume more products favoring overweight when they are together than when they are sitting alone at home.

### Limitations and Strengths

We analyzed the overall PA time and out-of-school sitting time of adolescents. However, our data did not provide information on the quantity of MVPA, and therefore we could not determine whether the adolescents fulfilled the international recommendations for MVPA. Moreover, the questionnaire did not elicit information on the type of the activities they were doing. It would be helpful and even necessary that future studies include further investigation about the adolescents' activities.

The current study collected information from adolescents *via* a self-administered survey that allowed them to self-assess their PA. While this method is widely used worldwide to collect PA information, it is influenced by the ability to make fairly accurate assessments. We acknowledge that an objective dataset collected from activity sensors or accelerometers would provide a more reliable assessment of PA in New Caledonia across ages, genders, and ethnicities (84).

The two IOTF weight status categories, underweight and normal, were pooled together even though underweight is not considered healthy. We chose to do so because of the low proportion of underweight adolescents (females in rural areas: 6 Melanesians, 2 Caucasians, and 0 Polynesians; females in urban areas: 1 Melanesian, 7 Caucasians, and 2 Polynesians; males in rural areas: 3 Melanesians, 3 Caucasians, and 0 Polynesians; males in urban areas: 1 Melanesian, 3 Caucasians, and 1 Polynesian). In addition, we found no significant difference between these categories for PA or sitting time.

This is a cross-sectional study so it cannot provide evidence of causal relationships between the factors we looked at and PA or these factors and weight status. But to our knowledge, this is the only study thus far in New Caledonia reporting data on PA and sitting time for adolescents.

Last, we acknowledge that the sample size for the Polynesian adolescents was rather small. We were not able to draw consistent conclusions about this population but we found relevant results by comparing the two largest ethnic communities, i.e., Melanesians and Caucasians.

### Considerations and Implications of the Study

As noted in a study conducted in Vanuatu, traditional gardening should be considered an important protective factor against obesity in the Melanesian culture (31). The current study did not provide corroborating evidence for this because we did not specifically study how the adolescents engaged in PA. However, we found that the Melanesian adolescents living in rural environments were physically more active than those living in the urban environment, probably because in the Melanesian tribal lifestyle people regularly engage in such community activities as gardening, hunting and fishing. Adolescents are thus generally involved in these activities with extended family and friends (16, 30). Despite the lifestyle transition occurring in New Caledonia and other PICTs, promoting outdoor activities and traditional foods, especially for rural Melanesians (39), might encourage the population to maintain healthy PA levels and limit unhealthy sedentary time. Providing spaces for leisure-time PA, such as sports, is another way to promote physically active behavior in adolescents, but Bauman et al. (5), who studied energy expenditures in young adults living in China, found this kind of PA insufficient to prevent obesity. The authors suggested that an “active living” lifestyle with active transport and occupational activity could help people reach a 60 min/day quantum of PA for obesity prevention and weight loss (5). Walking or cycling to school would be an interesting way to increase the PA levels in the adolescents of New Caledonia, especially those living in urban areas. Unfortunately, these modes of transport seem unsuitable for many rural adolescents whose schools are often located at a fair distance from home. PA promotion could alternatively make these adolescents more aware of outdoor activities instead of spending too much time in sedentary activities.

## Conclusion

This study, the first of this type in New Caledonia, shows that although 66% of adolescents were physically active, notable disparities existed in relation to place of residence, gender and ethnicity. Girls were less active on average than boys only for Melanesian adolescents living in rural areas. Melanesian adolescent boys living in rural areas were the most active group, suggesting that, in rural areas, activities related to the traditional Melanesian lifestyle may help adolescents to be active. In addition, feeling safe in the community was also identified as an important driver for PA. Although physically active adolescent boys were less likely to be overweight or obese, this was not the case for adolescent girls, perhaps because the girls engaged in different types of PA compared to boys. Additional research is warranted to better understand the barriers and facilitators driving overall engagement in PA and the role of PA and exercise on metabolic health in New Caledonian adolescents.

## Data Availability Statement

The raw data supporting the conclusions of this article will be made available by the authors, without undue reservation.

## Ethics Statement

The studies involving human participants were reviewed and approved by Human Research Ethics Committee of the University of New Caledonia. Written informed consent to participate in this study was provided by the participants' legal guardian/next of kin.

## Author Contributions

SF and OG conceived and designed the study. SF, CC, and OG collected data. GW conducted the statistical analyses and drafted the manuscript. CC, OG, and GW interpreted the results. All the authors participated in writing the manuscript, revised, and approved its final submitted version.

## Conflict of Interest

The authors declare that the research was conducted in the absence of any commercial or financial relationships that could be construed as a potential conflict of interest.

## Publisher's Note

All claims expressed in this article are solely those of the authors and do not necessarily represent those of their affiliated organizations, or those of the publisher, the editors and the reviewers. Any product that may be evaluated in this article, or claim that may be made by its manufacturer, is not guaranteed or endorsed by the publisher.
