# Supplementary material for: Physical Activity in Adolescents Living in Rural and Urban New Caledonia: The Role of Socioenvironmental Factors and the Association With Weight Status
Source: Front Public Health. 2021 Aug 6;9:623685. doi: 10.3389/fpubh.2021.623685 (PMC8378254; doi:10.3389/fpubh.2021.623685)
Supplement: Supplementary file 1 [file Table_1.pdf]

frontiers

Physical activity in adolescents living in rural and urban New Caledonia: the role of socioenvironmental factors and the association with weight status

Supplementary Material

Table S1. Anthropometric, sociodemographic characteristics and physical activities of the female adolescents according to ethnic community and place of living. Numbers indicate "Mean ± Standard deviation" for the numeric factors (noted with [m]) and "Size (%)" for the categorical factors (noted with [p]).

		Melanesian				Caucasian				Polynesian			
		All	Rural	Urban	p-value	All	Rural	Urban	p-value	All	Rural	Urban	p-value
	Number of participants	176	161	15		91	55	36		15	5	10	
	Age (years) [m]	13.44 ± 1.21	13.37 ± 1.18	14.17 ± 1.31*	0.013	13.83 ± 1.42	13.30 ± 1.24	14.64 ± 1.31	< 0.001	13.46 ± 1.86	13.18 ± 1.78	13.6 ± 1.98	0.698
SES	Low [p]	101 (57.39)	98 (60.87)	3 (20.00)		30 (43.96)	24 (43.64)	6 (16.67)		4 (26.67)	3 (60.00)	1 (10.00)	
	Intermediate [p]	43 (24.43)	36 (22.36)	7 (46.67)	0.005	21 (23.08)	10 (18.18) *	11 (30.56)	0.026	5 (33.33)	0 (0.00)	5 (50.00)	0.096
	High [p]	32 (18.18)	27 (16.77)	5 (33.33)		40 (43.96)	21 (38.18)	19 (52.78)		6 (40.00)	2 (40.00)	4 (40.00)	
Anthropometry	Height (m) [m]	1.57 ± 0.07	1.56 ± 0.07	1.59 ± 0.07	0.122	1.58 ± 0.08	1.57 ± 0.08	1.61 ± 0.07*	0.018	1.59 ± 0.08	1.56 ± 0.07	1.6 ± 0.09	0.398
	Mass (kg) [m]	55.95 ± 13.41	56.37 ± 13.75	51.43 ± 7.83	0.042	54.86 ± 17.14	56.8 ± 19.84	51.9 ± 11.57	0.141	61.35 ± 17.15	60.86 ± 18.47	61.60 ± 17.48	0.941
	BMI (kg/m²) [m]	22.67 ± 4.72	22.89 ± 4.79	20.29 ± 3.01	0.041	21.75 ± 5.84	22.91 ± 6.84	19.97 ± 3.19	0.041	24.00 ± 5.25	24.71 ± 6.00	23.65 ± 5.14	0.726
	IOTF BMI z-score [m]	0.97 ± 1.06	1.03 ± 1.05	0.24 ± 0.98	0.006	0.55 ± 1.23	0.89 ± 1.25	0.02 ± 1.01	0.001	1.21 ± 1.35	1.41 ± 1.34	1.11 ± 1.41	0.692
IOTF weight status	Not overweight [p]	101 (57.39)	90 (55.90)	11 (73.33)	0.302	68 (74.73)	36 (65.45)	32 (88.89)	0.023	6 (40.00)	1 (20.00)	5 (50.00)	0.580
	Overweight [p]	75 (42.61)	71 (44.10)	4 (26.67)		23 (25.27)	19 (34.55)	4 (11.11)		9 (60.00)	4 (80.00)	5 (50.00)	
Physical activity and sitting time	PA (min/day) [m]	113 ± 64	115 ± 63*	88 ± 75	0.118	85 ± 67	93 ± 75	72 ± 49	0.462	73 ± 43	75 ± 64	71 ± 31	0.862
	Out-of-school sitting time (min/day) [m]	164 ± 158	166 ± 158	156 ± 166	0.826	295 ± 218	271 ± 218	332 ± 217	0.144	152 ± 134	96 ± 53	180 ± 155	0.331
	PA ≥ 60 min/day [p]	129 (73.30)	122 (75.78)	7 (46.67)	0.028	48 (52.45)	29 (52.73)	19 (52.78)	1.000	9 (60.00)	3 (60.00)	6 (60.00)	1.000
	Out-of-school sitting time ≥ 120 min/day [p]	73 (41.48)	66 (40.99)	7 (46.67)	0.879	62 (68.13)	33 (60.00)	29 (80.56)	0.068	8 (53.33)	2 (40.00)	6 (60.00)	0.326
Socio-environmental factors	Siblings [m]	3.9 ± 2.8	3.9 ± 2.8	3.9 ± 2.7	0.885	2.1 ± 1.6	2.3 ± 1.7	1.9 ± 1.3	0.679	2.6 ± 1.5	3.2 ± 1.6	2.3 ± 1.5	0.288
	Peers [p]	107 (60.80)	101 (62.73)	6 (40.00)	0.148	61 (67.03)	37 (67.27)	24 (66.67)	1.000	5 (33.33)	1 (20.00)	4 (40.00)	0.600
	Family [p]	121 (68.75)	111 (69.94)	10 (66.67)	1.000	50 (54.95)	34 (61.82)	16 (44.44)	0.158	10 (66.67)	2 (40.00)	8 (80.00)	0.251
	Safety of area [p]	111 (63.07)	103 (63.98)	8 (53.33) *	0.591	54 (59.34)	30 (54.55)	24 (66.67)	0.351	11 (73.33)	4 (80.00)	7 (70.00)	1.000
	Accessibility of area [p]	119 (67.61)	112 (69.57)	7 (46.67)	0.086	77 (84.62)	45 (81.82)	32 (88.89)	0.537	10 (66.67)	3 (60.00)	7 (70.00)	1.000

* Significant difference between boys and girls ($p < 0.05$).
[m] Numbers for this line are: Mean ± Standard deviation.
[p] Numbers for this line are: Size (%).
