# Supplementary material for: Physical Activity in Adolescents Living in Rural and Urban New Caledonia: The Role of Socioenvironmental Factors and the Association With Weight Status
Source: Front Public Health. 2021 Aug 6;9:623685. doi: 10.3389/fpubh.2021.623685 (PMC8378254; doi:10.3389/fpubh.2021.623685)
Supplement: Supplementary file 2 [file Table_2.pdf]

**Table S2. Anthropometric, sociodemographic characteristics and physical activities of the male adolescents according to ethnic community and place of living. Numbers indicate "Mean ± Standard deviation" for the numeric factors (noted with [m]) and "Size (%)" for the categorical factors (noted with [p]).**

| | | Melanesian | | | | Caucasian | | | | Polynesian | | | |
|---|---|---|---|---|---|---|---|---|---|---|---|---|---|
| | | All | Rural | Urban | p-value | All | Rural | Urban | p-value | All | Rural | Urban | p-value |
| | Number of participants | 143 | 119 | 24 | | 67 | 39 | 28 | | 16 | 5 | 11 | |
| | Age (years) [m] | 13.22 ± 1.25 | 13.25 ± 1.20 | 13.09 ± 1.47 | 0.571 | 13.55 ± 1.62 | 12.99 ± 1.28 | 14.33 ± 1.73 | 0.001 | 12.88 ± 1.82 | 12.97 ± 1.51 | 12.84 ± 2.01 | 0.903 |
| SES | Low [p] | 70 (48.95) | 64 (53.78) | 6 (25.00) | 0.032 | 14 (20.90) | 11 (28.21) | 3 (10.71) | 0.005 | 8 (50.00) | 3 (60.00) | 5 (45.45) | 1.000 |
| | Intermediate [p] | 40 (27.97) | 31 (26.05) | 9 (37.50) | | 23 (34.33) | 17 (43.59) | 6 (21.43) | | 4 (25.00) | 1 (20.00) | 3 (27.27) | |
| | High [p] | 33 (23.08) | 24 (20.17) | 9 (37.50) | | 30 (44.78) | 11 (28.21) | 19 (67.86) | | 4 (25.00) | 1 (20.00) | 3 (27.27) | |
| Anthropometry | Height (m) [m] | 1.56 ± 0.09 | 1.56 ± 0.09 | 1.57 ± 0.12 | 0.769 | 1.60 ± 0.12 | 1.55 ± 0.10 | 1.65 ± 0.11 | < 0.001 | 1.63 ± 0.14 | 1.61 ± 0.21 | 1.63 ± 0.11 | 0.822 |
| | Mass (kg) [m] | 54.28 ± 16.18 | 54.06 ± 15.67 | 55.35 ± 18.85 | 0.766 | 51.98 ± 15.05 | 51.18 ± 16.51 | 53.09 ± 12.96 | 0.340 | 64.36 ± 21.19 | 69.56 ± 30.48 | 62.00 ± 16.80 | 0.527 |
| | BMI (kg/m²) [m] | 22.01 ± 5.29 | 21.95 ± 5.07 | 22.27 ± 6.4 | 0.985 | 20.14 ± 4.48 | 20.79 ± 5.05 | 19.23 ± 3.43 | 0.353 | 23.96 ± 6.69 | 25.80 ± 8.50 | 23.13 ± 5.98 | 0.478 |
| | IOTF BMI z-score [m] | 0.95 ± 1.20 | 0.95 ± 1.18 | 0.96 ± 1.32 | 0.962 | 0.37 ± 1.25 | 0.64 ± 1.25 | -0.02 ± 1.16 | 0.031 | 1.31 ± 1.50 | 1.64 ± 1.47 | 1.16 ± 1.56 | 0.567 |
| IOTF weight status | Not overweight [p] | 89 (62.24) | 74 (62.18) | 15 (62.50) | 1.000 | 53 (79.10) | 28 (71.79) | 25 (89.29) | 0.152 | 7 (43.75) | 1 (20.00) | 6 (54.54) | 0.308 |
| | Overweight [p] | 54 (37.76) | 45 (37.82) | 9 (37.50) | | 14 (20.90) | 11 (28.21) | 3 (10.71) | | 9 (56.25) | 4 (80.00) | 5 (45.45) | |
| Physical activity and sitting time | PA (min/day) [m] | 127 ± 81 | 133 ± 80 | 95 ± 79 | 0.028 | 101 ± 76 | 97 ± 81 | 108 ± 68 | 0.239 | 80 ± 52 | 69 ± 44 | 86 ± 57 | 0.561 |
| | Out-of-school sitting time (min/day) [m] | 167 ± 166 | 170 ± 171 | 152 ± 144 | 0.862 | 239 ± 166 | 231 ± 152 | 251 ± 185 | 0.898 | 229 ± 205 | 180 ± 127 | 251 ± 233 | 0.818 |
| | PA ≥ 60 min/day [p] | 100 (69.93) | 89 (74.79) | 11 (45.83) | 0.010 | 42 (62.69) | 25 (64.10) | 17 (60.71) | 0.979 | 8 (50.00) | 2 (40.00) | 6 (54.55) | 1.000 |
| | Out-of-school sitting time ≥ 120 min/day [p] | 62 (43.36) | 51 (42.86) | 11 (45.83) | 0.966 | 50 (74.63) | 28 (71.79) | 22 (78.57) | 0.731 | 12 (75.00) | 3 (60.00) | 9 (81.82) | 0.547 |
| Socio-environmental factors | Siblings [m] | 3.3 ± 2.4 | 3.4 ± 2.5 | 2.3 ± 1.7 | 0.016 | 2.1 ± 1.5 | 2.6 ± 1.7 | 1.4 ± 0.7 | 0.001 | 2.6 ± 1.3 | 2.6 ± 0.5 | 2.5 ± 1.5 | 1.000 |
| | Peers [p] | 89 (62.24) | 76 (63.87) | 13 (54.17) | 0.507 | 42 (62.69) | 25 (64.10) | 17 (60.71) | 0.979 | 10 (62.5) | 2 (40.00) | 8 (72.73) | 0.300 |
| | Family [p] | 83 (58.04) | 73 (61.34) | 10 (41.67) | 0.120 | 29 (43.28) | 19 (48.72) | 10 (35.71) | 0.418 | 8 (50.00) | 3 (60.00) | 5 (45.45) | 1.000 |
| | Safety of area [p] | 104 (72.73) | 83 (69.75) | 21 (87.50) | 0.126 | 40 (59.70) | 21 (53.85) | 19 (67.86) | 0.368 | 7 (43.75) | 2 (40.00) | 5 (45.45) | 1.000 |
| | Accessibility of area [p] | 109 (76.22) | 91 (76.47) | 18 (75.00) | 1.000 | 57 (85.07) | 33 (84.62) | 24 (85.71) | 1.000 | 8 (50.00) | 4 (80.00) | 4 (36.36) | 0.282 |

[m] Numbers for this line are: Mean ± Standard deviation.

[p] Numbers for this line are: Size (%).
