# Supplementary material for: Physical Activity in Adolescents Living in Rural and Urban New Caledonia: The Role of Socioenvironmental Factors and the Association With Weight Status
Source: Front Public Health. 2021 Aug 6;9:623685. doi: 10.3389/fpubh.2021.623685 (PMC8378254; doi:10.3389/fpubh.2021.623685)
Supplement: Supplementary file 3 [file Table_3.pdf]

Table S3. P-values of overall tests (One-way ANOVA or Kruskal-Wallis test for numeric variables – noted with ^{m} – and χ^2 or Fisher's exact test for categorical variables – noted with ^{p}) and post-hoc tests for ethnic community comparison according to place of living in female adolescents.

	All				Rural				Urban			
	Overall test*	Post-hoc test			Overall test*	Post-hoc test			Overall test*	Post-hoc test		
		M-C	M-P	C-P		M-C	M-P	C-P		M-C	M-P	C-P
Age (years) ^{m}	0.067^{S}				0.890^{S}				0.119^{M}			
SES ^{p}	< 0.001^{M}	< 0.001	0.116	1.000	0.011^{M}	0.019	0.603	1.000	0.640^{S}			
Height (m) ^{m}	0.208^{S}				0.975^{S}				0.872^{S}			
Mass (kg) ^{m}	0.050^{S}				0.388^{S}				0.067^{M}			
BMI (kg/m²) ^{m}	0.184^{S}				0.289^{S}				0.017^{M}	0.951	0.058	0.013
IOTF BMI z-score ^{m}	0.008^{S}	0.011	0.694	0.086	0.504^{S}				0.023^{M}	0.773	0.127	0.017
IOTF weight status ^{p}	0.004^{S}	0.024	0.907	0.038	0.107^{S}				0.025^{M}	0.638	1.000	0.045
PA (min/day) ^{m}	< 0.001^{S}	< 0.001	0.060	0.977	0.014^{S}	0.018	0.406	0.880	0.933^{S}			
Out-of-school sitting time ^{m}	< 0.001^{M}	< 0.001	0.941	0.074	0.008^{S}	0.008	0.854	0.164	0.010^{M}	0.019	0.735	0.119
PA ≥ 60 min/day ^{p}	0.003^{M}	0.003	1.000	1.000	0.005^{M}	0.006	1.000	1.000	0.826^{S}			
Out-of-school sitting time ≥ 120 min/day ^{p}	< 0.001^{S}	< 0.001	1.00	1.000	0.046^{S}	0.067	1.000	1.000	0.046^{M}	0.067	1.000	0.659
Siblings ^{m}	< 0.001^{M}	< 0.001	0.280	0.290	< 0.001^{M}	< 0.001	0.960	0.340	0.039^{M}	0.032	0.507	0.602
Peers ^{p}	0.044^{M}	1.000	0.216	0.082	0.116^{S}				0.123^{M}			
Family ^{p}	0.081^{S}				0.255^{S}				0.099^{M}			
Safety of area ^{p}	0.560^{S}				0.355^{S}				0.701^{S}			
Accessibility of area ^{p}	0.007^{S}	0.010	1.000	0.417	0.131^{S}				0.004^{L}	0.008	1.000	0.490

In the "Post-hoc test" columns: "M-C" sub-columns mean comparison between Melanesians and Caucasians, "M-P" sub-columns mean comparison between Melanesians and Polynesians and "C-P" sub-columns mean comparison between Caucasians and Polynesians.

^{m} Numerical factors.
^{p} Categorical factors.
* Superscript letters inform about the effect size magnitude: small (S), moderate (M) and large (L).
