# Supplementary material for: Physical Activity in Adolescents Living in Rural and Urban New Caledonia: The Role of Socioenvironmental Factors and the Association With Weight Status
Source: Front Public Health. 2021 Aug 6;9:623685. doi: 10.3389/fpubh.2021.623685 (PMC8378254; doi:10.3389/fpubh.2021.623685)
Supplement: Supplementary file 4 [file Table_4.pdf]

Table S4. P-values of overall tests (One-way ANOVA or Kruskal-Wallis test for numeric variables – noted with [m] – and χ^2 or Fisher's exact test for categorical variables– noted with [p]) and post-hoc tests for ethnic community comparison according to place of living in male adolescents.

	All				Rural				Urban			
	Overall test*	Post-hoc test			Overall test*	Post-hoc test			Overall test*	Post-hoc test		
		M-C	M-P	C-P		M-C	M-P	C-P		M-C	M-P	C-P
Age (years) [m]	0.208[S]				0.487[S]				0.011[M]	0.027	0.915	0.041
SES [p]	0.001[M]	0.001	1.000	0.204	0.051[M]				0.057[L]			
Height (m) [m]	0.023[S]	0.092	0.083	0.676	0.860[S]				0.022[M]	0.018	0.250	0.844
Mass (kg) [m]	0.025[S]	0.594	0.048	0.017	0.063[S]				0.305[S]			
BMI (kg/m²) [m]	0.009[S]	0.038	0.313	0.021	0.105[S]				0.045[M]	0.096	0.891	0.095
IOTF BMI z-score [m]	0.002[S]	0.004	0.503	0.017	0.151[S]				0.009[L]	0.023	0.908	0.035
IOTF weight status [p]	0.007[S]	0.053	0.546	0.031	0.067[S]				0.024[M]	0.135	1.000	0.083
PA (min/day) [m]	0.017[S]	0.061	0.085	0.767	0.008[S]	0.018	0.164	0.953	0.614[S]			
Out-of-school sitting time [m]	0.001[S]	0.001	0.151	0.971	0.024[S]	0.019	0.830	0.755	0.085[S]			
PA ≥ 60 min/day [p]	0.208[S]				0.105[S]				0.562[S]			
Out-of-school sitting time ≥ 120 min/day [p]	< 0.001[M]	< 0.001	0.097	1.000	0.004[M]	0.009	1.000	1.000	0.030[M]	0.093	0.208	1.000
Siblings [m]	< 0.001[M]	< 0.001	0.637	0.168	0.072[S]				0.029[M]	0.110	0.800	0.040
Peers [p]	0.998[S]				0.577[S]				0.624[S]			
Family [p]	0.122[S]				0.379[S]				0.836[S]			
Safety of area [p]	0.022[S]	0.237	0.068	0.822	0.379[S]				0.032[M]	0.338	0.044	0.832
Accessibility of area [p]	0.013[S]	0.454	0.105	0.015	0.639[S]				0.010[L]	1.000	0.171	0.013

In the "Post-hoc test" columns: "M-C" sub-columns mean comparison between Melanesians and Caucasians, "M-P" sub-columns mean comparison between Melanesians and Polynesians and "C-P" sub-columns mean comparison between Caucasians and Polynesians.

[m] Numerical factors.
[p] Categorical factors.
[*] Superscript letters inform about the effect size magnitude: small (S), moderate (M) and large (L).
